# The Evaluation of Risk Factors for Postoperative Infectious Complications after Percutaneous Nephrolithotomy

**DOI:** 10.1155/2017/4832051

**Published:** 2017-02-02

**Authors:** Tian Yang, Shenghua Liu, Jimeng Hu, Lujia Wang, Haowen Jiang

**Affiliations:** ^1^Fudan Institute of Urology, Huashan Hospital, Fudan University, Shanghai, China; ^2^Department of Urology, Huashan Hospital, Fudan University, Shanghai, China

## Abstract

This study was to evaluate the risk factors of infectious complications after percutaneous nephrolithotomy (PCNL) and build a prediction tool for postoperative complications based on the risk factors. A total of 110 male (67.1%) and 54 female (32.9%) patients who underwent PCNL for renal stones between 2010 and 2014 in our institute were included. A detailed clinical information and laboratory results were obtained from patients. Systemic inflammatory response syndrome (SIRS) and postoperative fever were recorded after PCNL surgery. In all, 45 cases (27.4%) developed SIRS and fever was observed in 20 cases (12.2%). In multivariate analysis, stone size (odds ratio, OR = 1.471, *p* = 0.009) and urine white blood cell (WBC) (OR = 1.001, *p* = 0.007) were related to the development of SIRS. Stone size (OR = 1.644, *p* = 0.024), urine WBC (OR = 1.001, *p* = 0.002) and serum albumin (OR = 0.807, *p* = 0.021) were associated with postoperative fever. We concluded that patients with larger stone size and preoperative urinary tract infection might have a higher risk of developing SIRS and fever after operation, while a high-normal level of serum albumin might be the protective factor for postoperative fever.

## 1. Introduction

Urolithiasis is one of the most common benign urologic diseases, with a nearly 10% of lifetime incidence [1]. In addition, the prevalence of urolithiasis has been rising through the decade worldwide [2]. In the United States, the prevalence rate increased from 3.8% in the 1970s to 8.8% in the 2000s [3].

Several types of urolithiasis have been identified of which staghorn calculi and large renal calculi are of clinical importance. These two types of calculi may cause the damage to renal function. Besides, they are difficult to be treated with regular ureteroscopic procedures. Percutaneous nephrolithotomy (PCNL) is the standard treatment for large renal stones >2 cm and staghorn calculi. It is less traumatic and quicker recovery compared with open surgery. In addition, PCNL has a higher rate of stone clearance than extracorporeal shock wave lithotripsy [4]. However, relatively higher perioperative complication rates were also reported [5] including fever (10.8%), blood transfusion (7%), thoracic complications (1.5%), sepsis (0.5%), embolization (0.4%), organ injury (0.4%), and urinoma (0.2%). Comorbidity such as renal insufficiency, diabetes, obesity, and pulmonary disease will increase the risk of complications [6]. In several studies, a 0.3% of 30-day mortality rate of PCNL was reported [7]. The majority of death cases were caused by septicemia after surgery. Preoperative risk factors that will increase the complication rates were observed; for instance, a large size of stone, staghorn calculi, and hydronephrosis were the risk factors of postoperative SIRS [8]. However, these studies mainly focused on factors of lithiasis instead of other clinical information, such as results of urine routine testing, and use of antibiotics.

In this study, our objective was to evaluate the association between preoperative risk factors and post-PCNL complications and establish a clinical risk prediction tool for post-PCNL complications.

## 2. Materials and Methods

All patients who underwent PCNL for renal stones from 2010 to 2014, in Huashan Hospital, Fudan University, Shanghai, China, were included in this study. The operations were all performed by three experienced urologists following standard procedure. Detailed clinical information was obtained retrospectively, including the stone size, stone type, number of stones, hydronephrosis, surgery time, days of therapeutic antibiotics use before surgery, and blood transfusion during surgery. Postoperative conditions were recorded including body temperatures and hospital stay. Laboratory investigations were conducted in all the patients included blood routine, urine routine, liver function, and renal function tests before and after surgery. Urine culture was studied in patients with preoperation urine WBC (+) in order to guide the use of antibiotic after operation. Besides, anesthetists would also assess the systemic condition of each patient before surgery to ensure the operation indication. As PCNL was a selective operation, patients with other focus of infection would not undergo the surgery until the infection was controlled. In those patients, different antibiotics (e.g., levofloxacin, tazocilline, and meropenem) were used according to the severity of infection and patients' allergic history. Such antibiotic would also be used prophylactically before surgery. For patients without postoperation infectious complications, they would receive one dose of preventive cefuroxime 30 minutes before surgery.

SIRS criteria were identified according to the census statement published by the American College of Chest Physicians (ACCP) and the Society of Critical Care Medicine (SCCM) in 2001 (two or more of the following): (1) body temperature > 38°C or < 36°C; (2) heart rate > 90 bpm; (3) respiratory rate > 20 breaths/min or PaCO_2_ < 32 mmHg; (4) white blood cell count > 12,000 cells/*μ*L or < 4,000 cells/*μ* [9]. Postoperative fever with clinical significance was defined as body temperature above 38.5°C within hospital stay. Time of hospital stay was defined as the number of days after surgery. The study was approved by institutional review board of Huashan Hospital, Fudan University, Shanghai, China. All the methods used in this study were carried out in accordance with the approved guidelines from the above board and the principles of Helsinki Declaration were followed. Written informed consent was obtained from each patient.

All patients were categorized into groups according to postoperative SIRS or fever status. Chi-square test was used to compare categorical variables. Student's *t*-test was used to compare the means of continuous variables between groups. Variables that showed significant differences were included in a multivariate logistic regression analysis. A model based on the multivariate logistic regression results was set up to predict postoperative SIRS. Statistical analyses were performed using the SPSS version 19.0 (SPSS Inc., Chicago, IL, USA). Two-tailed *p* values of <0.05 were considered statistically significant.

## 3. Results

A total of 164 patients were finally included in this study, with 110 males (67.1%) and 54 females (32.9%). Baseline characteristics of study population were shown in Table 1. The mean age was 51.07 ± 12.08. Forty-five cases (27.4%) of SIRS, 20 cases (12.2%) of fever, and 1 (0.61%) case of infectious shock were observed after surgery.

**Figure 1:**
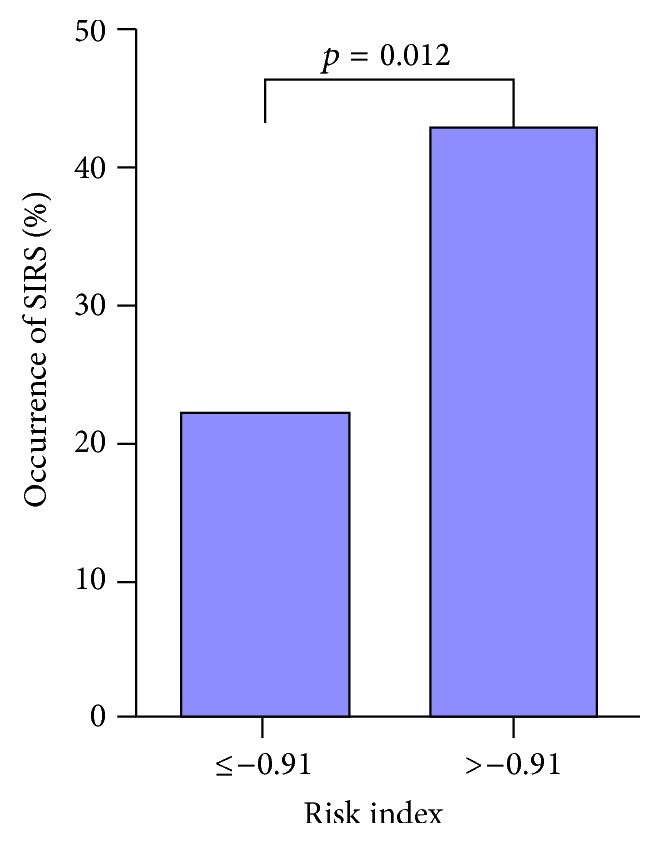
The predictive value of the occurrence of SIRS based on 2 risk indexes (≤−0.91 and >−0.91).

Stone size (*p* = 0.012) and urine WBC (*p* = 0.001) were significantly associated with postoperative SIRS, which was shown in Table 2. In the multivariate logistic regression analysis, stone size [OR = 1.471, 95% confidence interval (CI) = 1.098–1.972, *p* = 0.009] and urine WBC [OR = 1.001, 95% (CI) = 1.000–1.002, *p* = 0.007] were found independently associated with postoperative SIRS. (Table 3). A prediction model was built based on these two risk factors. By using the model, a risk index would be calculated (risk index = 0.001 *∗* urine WBC (/*μ*L) + 0.389 *∗* stone size (cm) − 2.325). We then categorized patients into two groups according to the risk index from the prediction model (≤−0.91, >−0.91). We observed that patients with a risk index of over −0.91 would have 42.86% probability of having postoperative SIRS, almost twice higher than patients with a lower (22.22%) risk index (*p* = 0.012) (Figure 1).

Since fever is the most common precursory symptom of SIRS, we then investigated the risk factors of postoperative fever. We observed that postoperative fever was significantly associated with stone size (*p* = 0.016), urine WBC (*p* < 0.001), and serum albumin (*p* = 0.018) in univariate analysis (Table 4), while, in multivariate analysis, only stone size [OR = 1.644, 95% confidence interval (CI) = 1.068–2.531, *p* = 0.024], urine WBC [OR = 1.001, 95% (CI) = 1.000–1.002, *p* = 0.002], and serum albumin [OR = 0.807, 95% (CI) = 0.673–0.968, *p* = 0.021] remained statistically significant (Table 5).

## 4. Discussion

PCNL is one of the most important choices for urologists to treat renal stones with hydronephrosis. However, with relatively high complication rates and fatal complication rates (e.g., sepsis and septic shock), the application of PCNL was limited. Studies had reported that stone size, hydronephrosis, staghorn calculi, paraplegia, stone in calyceal location, blood transfusion, and a previous PCNL were risk factors of postoperative SIRS. Female gender, positive preoperative urine culture, stone size, preoperative nephrostomy, operative time, and a positive stone culture were risk factors of postoperative fever. However, the results varied in different studies [10–13]. In the current study, we evaluated the potential risk factors of postoperative complications and observed that urine WBC and stone size were two risk factors for both fever and SIRS, while a high-normal level of serum albumin was the protective factor of postoperative fever.

In our study, the postoperative complication rates were 12.2% for fever and 27.4% for SIRS, respectively. The SIRS rate is similar to previous studies [8, 10, 14]. The occurrence rate of fever is 2.8–32.1% according to a study in a large European population [7]. The wide variation of the postoperative fever occurring rate across different studies may be due to the following reasons. First, the criteria of fever were different in various studies. In our study, postoperative fever was defined as body temperature above 38.5°C within hospital stay, while others have used a temperature of ≥38.0°C [15, 16]. Secondly, the time between PCNL and the assessment of postoperative temperature may also influence the fever occurrence rate reported in different studies. Draga et al. found that though 39.8% of patients had a fever in the first 24 hours after PCNL, the rate decreased to only 13.0% when assessed beyond 24 hours [16]. Thus, we considered the results of the current study were comparable with other studies.

The elevation of urine WBC before surgery and the stone size were found significantly associated with the development of both SIRS and fever, which was similar to other studies [11–13, 16, 17]. Studies showed that patients with recurrent urinary tract infections had a higher risk of developing SIRS after PCNL [16]. Besides, existing urinary tract infection would increase the risk of postoperative fever [17]. Urinary tract infection was commonly seen in patients with renal stones, especially in those who have infection calculi (struvite). Studies showed that bacteria grow slowly in infection calculi and form a biofilm which makes the antibiotics difficult to kill them thoroughly [18]. During the surgical procedure, the bacteria and endotoxin will be released, which may cause SIRS and fever. Stone with larger size will lead to urinary tract obstruction and increase the intracavity pressure above the obstruction. Besides, larger stone increases the difficulty of surgical procedures, which may prolong the time of operation and time of iatrogenic hydronephrosis during the surgery. This may increase the probability of retrograde infection via operation. Therefore, it is necessary to complete the urine routine test and urine culture examination before surgery in order to evaluate the severity of urinary tract infection. The use of preoperative antibiotics before surgery is also suggested, especially for patients who have larger stone. PCNL should be conducted after infection is under control.

Interestingly, a high-normal level of serum albumin before surgery was found to be a protective factor for post-operation fever in our study, which was seldom reported in other studies. Studies reported that in febrile patients, a lower serum albumin level is a predictive factor for microbial infection [19]. It may explain the results in our study. A lower level of albumin would cause the insufficient synthesis of immunoglobulin, which might weaken the immune system. Thus, patients were more likely to be infected by bacteria or virus, causing fever and SIRS. However, in our study, we did not observe a significant association between serum albumin and SIRS. Further evaluation is needed in a larger scale of population.

In the current study, we hoped to establish a prediction tool for the occurrence of SIRS after PCNL. The common prediction factors are urine culture and stone type. Since the urine culture result needed a long time and some of the medical centers could not carry out the stone analysis, we chose to establish a risk prediction model based on the available information before operation from the multivariate logistic regression result. In the current study, stone size and urine WBC were found significant in the multivariate analysis after adjusting for other variables. So our model was built based on these two variables. The merit of the prediction model was that it was all based on available clinical information, so it could offer a quick and convenient evidence for further clinical treatment.

In the previous studies, we observed that there were some differences in the proportion of stone composition between Chinese population and American population. In China, oxalate (86.7%) was the most common type followed by uric acid (5.1%), hydroxyapatite (5%), struvite (3%), and cystine (0.2%). While in America, a study from Mayo Clinic Metals Laboratory (*n* = 43935) showed that oxalate (67%) was the most common type followed by hydroxyapatite (16%), uric acid (8%), struvite (3%), brushite (0.9%), and cystine (0.35%) [20]. China owns more patients with oxalate stone and less with uric acid stone. However, the relationship between stone composition and incidence of postoperative infection had not been assessed yet. It seems that struvite and apatite stones carry a particularly high risk of infection after PCNL. In a study of 34 renal calculi, 94% of the infected calculi were composed of struvite or apatite and the levels of endotoxin in these stones were approximately 40 times higher than that of uninfected stones (calcium oxalate, uric acid, and cystine stones) [21].

In our study, one patient developed septic shock 2 days after surgery and was saved after emergent antishock therapy. The patients recovered well and left hospital on the 6th day after surgery. Reviewing his history, this patient had multiple stones of 3 cm in diameter and the urine WBC count before surgery was 2377/*μ*L. It conformed to our results that patients with larger stone and higher urine WBC count are more likely to develop postoperative SIRS and fever.

There remained several limitations in our study. First, this is a retrospective study from single institute, which might lead to selection bias. However, Huashan Hospital is a tertiary hospital in Shanghai, China. Patients from all over the country seek their healthcare service because of its high quality. Therefore, patients in our institute may partly represent the Chinese population, especially population from southeast part of China. Secondly, with limited information of urine culture and blood culture for each patient in the current study, it is hard to provide an evaluation between postoperative complications and pathogens. Thirdly, when describing the relationship between serum albumin and postoperative fever, we failed to exclude interference factors such as nutrition disorders or liver diseases in our study. We did not find differences in terms of comorbid diseases between patients with low albumin levels and patients with normal levels, which may due to small sample of our study. Thus, it is difficult for us to clarify the cause-effect relationship between serum albumin level and postoperative fever, which is worth further investigation. Fourthly, as this is a retrospective study, most of the patients did not receive a stone composition analysis, which made it difficult for us to provide a more comprehensive analysis. The prediction tool could be more precise if we had the results of urine culture and stone analysis. Finally, with a relatively small sample size, rare complications (e.g., thoracic complications, organ injury, urinoma, and death) might not be observed. Large-scale prospective studies are needed for further evaluation.

## 5. Conclusions

In conclusion, we observed in our study that urine WBC and stone size were risk factors of both fever and SIRS, while a high-normal level of serum albumin might be a protective factor of fever. The prediction model including two risk factors might have added value for more appropriate perioperative care.

## Figures and Tables

**Table 1:** Clinical characteristics of study population.

Parameter	Mean (SD^*∗*^)
Age (year)	51.07 (12.08)
Stone size (cm)^†^	2.88 (1.30)
Surgery time (min)	133.66 (53.35)
Day of antibiotic use before surgery (days)	0.54 (1.44)

	*N* (%)

Gender	
Male	110 (67.1)
Female	54 (32.9)
Stone number^‡^	
<3	101 (61.6)
≥3	63 (38.4)
Diabetes	14 (8.5)
Staghorn calculi	7 (4.3)
Hydronephrosis	125 (76.2)
SIRS^§^	45 (27.4)
Postoperative fever	20 (12.2)
Shock	1 (0.61)

^†^Stone size: the measurement of diameter of the largest stone based on preoperative CT scanning.

^‡^Stone number: the total counting of the stone based on preoperative CT scanning.

^§^SIRS: systemic inflammatory response syndrome.

^*∗*^SD: standard deviation.

**Table 2:** Comparison of clinical factors between patients with and without postoperative SIRS.

	SIRS	No SIRS	*p*
*Continuous variables*			
Age (year): mean ± SD	53.13 ± 12.61	50.29 ± 11.84	0.179
Stone size (cm): mean ± SD	3.30 ± 1.45	2.72 ± 1.20	**0.012**
Day of antibiotic use before surgery (day): mean ± SD	0.51 ± 1.69	0.55 ± 1.34	0.863
Surgery time (min): mean ± SD	133.41 ± 52.47	133.75 ± 53.90	0.971
Temperature before Surgery: mean ± SD	36.96 ± 0.22	36.96 ± 0.21	0.081
Blood WBC (*∗*10^9^/L): ^†^Mean ± SD	6.47 ± 1.83	6.07 ± 1.99	0.254
Hemoglobin (g/L): mean ± SD	138.23 ± 14.50	134.39 ± 24.26	0.329
Urine WBC (/*μ*L)^‡^: mean ± SD	501.14 ± 994.92	131.44 ± 347.93	**0.001**
Serum albumin (g/L): mean ± SD	41.25 ± 3.62	41.54 ± 3.58	0.641
Serum creatinine (*μ*mol/L): mean ± SD	78.58 ± 24.94	79.32 ± 24.81	0.868
*Categorical variables*			
Gender			
Male	31	79	0.761
Female	14	40	
Stone number			
<3	29	72	0.643
≥3	16	47	
Staghorn calculi			
No	43	114	0.945
Yes	2	5	
Hydronephrosis			
No	7	32	0.128
Yes	38	87	
Diabetes			
No	40	110	0.468
Yes	5	9	
Blood transfusion			
No	42	112	0.888
Yes	2	6	

^†^Blood WBC: blood white blood cell count that was examined the day before surgery.

^‡^Urine WBC: urine white blood cell count that was examined the day before surgery.

**Table 3:** Multivariate analysis of factors between patients with and without postoperative SIRS.

	Odds ratio	*p*	Lower CI^†^	Upper CI
Stone size	1.471	**0.009**	1.098	1.972
Urine WBC	1.001	**0.007**	1.000	1.002
Temperature before surgery	0.237	0.114	0.040	1.414

^†^CI: confidence interval.

**Table 4:** Comparison of clinical factors between patients with and without postoperative fever.

	Fever	No fever	*p*
*Continuous variables*			
Age (year): mean ± SD	53.65 ± 11.31	50.70 ± 12.18	0.083
Stone size (cm): Mean ± SD	3.55 ± 1.39	2.79 ± 1.26	**0.016**
Day of antibiotic use before surgery (day): mean ± SD	0.60 ± 1.60	0.53 ± 1.42	0.850
Surgery time (min): Mean ± SD	128.75 ± 42.61	134.35 ± 54.79	0.662
Temperature before surgery: mean ± SD	36.89 ± 0.22	36.94 ± 0.22	0.754
Blood WBC (*∗*10^9^/L) ^a^mean ± SD	6.59 ± 2.14	6.13 ± 1.92	0.335
Hemoglobin (g/L): mean ± SD	134.42 ± 13.80	135.64 ± 22.88	0.822
Urine WBC (/*μ*L)^b^: mean ± SD	858.86 ± 1205.48	152.52 ± 444.81	<**0.001**
Serum albumin (g/L): mean ± SD	36.71 ± 3.72	47.32 ± 3.51	**0.018**
Serum creatinine (*μ*mol/L): mean ± SD	75.06 ± 17.87	79.65 ± 25.53	0.461
*Categorical variables*			
Gender			
Male	10	100	0.761
Female	10	44	
Stone number			
<3	11	90	0.518
≥3	9	54	
Staghorn calculi			
No	19	138	0.863
Yes	1	6	
Hydronephrosis			
No	5	34	0.891
Yes	15	110	
Diabetes			
No	18	132	0.803
Yes	2	12	
Blood transfusion			
No	20	134	0.276
Yes	0	8	

^a^Blood WBC, blood white blood cell count, that was examined the day before surgery. ^b^Urine WBC, urine white blood cell count, that was examined the day before surgery.

**Table 5:** Multivariate analysis of risk factors for postoperative fever.

	Odds ratio	*p*	Lower CI	Upper CI
Stone size	1.644	**0.024**	1.068	2.531
Urine WBC	1.001	**0.002**	1.000	1.002
Serum albumin	0.807	**0.021**	0.673	0.968
Age	1.025	0.383	0.970	1.084
